# 固相萃取-超高效液相色谱-串联质谱法测定人尿液中4种氧化应激生物标志物

**DOI:** 10.3724/SP.J.1123.2024.10003

**Published:** 2025-04-08

**Authors:** Zhuangzhuang FENG, Xiao LIN, Dejun BAO, Xiaojian HU, Haijing ZHANG, Ying ZHU, Xu ZHANG

**Affiliations:** 中国疾病预防控制中心环境与人群健康重点实验室,中国疾病预防控制中心环境与健康相关产品安全所,北京 100021; China CDC Key Laboratory of Environment and Population Health, National Institute of Environmental Health, Chinese Center for Disease Control and Prevention, Beijing 100021, China

**Keywords:** 固相萃取, 超高效液相色谱-串联质谱法, 氧化应激生物标志物, 尿液, 人体生物监测, solid-phase extraction (SPE), ultra performance liquid chromatography-tandem mass spectrometry (UPLC-MS/MS), oxidative stress biomarkers, urine, human biomonitoring

## Abstract

基于超高效液相色谱-串联质谱法(UPLC-MS/MS),建立了人尿液中8-羟基脱氧鸟苷(8-OHdG)、8-羟基鸟苷(8-OHG)、羟基壬烯巯基尿酸(HNEMA)和双酪氨酸(diY)等4种氧化应激生物标志物的测定方法。用水将0.2 mL尿样稀释5倍后,样品经Oasis HLB柱进行富集净化;先用2%(体积分数)甲醇水溶液洗脱diY,再用甲醇洗脱8-OHdG、8-OHG和HNEMA,收集洗脱液后分别进样分析;以甲醇和0.05%(体积分数)乙酸水溶液作为流动相进行梯度洗脱,Acquity UPLC HSS T3色谱柱进行色谱分离。分别在正离子电喷雾(ESI^+^)和负离子电喷雾(ESI^-^)多反应监测(MRM)模式下扫描化合物,同位素内标法定量。4种氧化应激生物标志物在0.01~100 μg/L范围内线性关系良好,相关系数均≥0.9998;检出限为7~18 ng/L,定量限为22~60 ng/L; 4种氧化应激标志物在低、中、高3个水平下的加标回收率分别为103.0%~105.6%(8-OHdG)、100.8%~104.2%(8-OHG)、97.2%~100.2%(diY)和96.9%~106.0%(HNEMA)。采用本方法测定40份尿液样本,结果表明8-OHdG、8-OHG、diY和HNEMA的检出率均为100%;检出质量浓度范围分别为0.52~14.40 μg/L、2.75~38.15 μg/L、8.92~82.28 μg/L和1.74~575.29 μg/L,中位值分别为2.89、12.36、37.66和96.92 μg/L。该方法操作简单,灵敏度高,精密度好,适用于开展人尿液中4种氧化应激生物标志物的测定。

## Abstract (en)

Oxidative stress biomarkers are measurable biological indicators that reflect the balance between the production of reactive oxygen species (ROS) and the body’s ability to neutralize them using antioxidants. Elevated oxidative stress is associated with a number of health effects. Herein, we report the development of a comprehensive and sensitive method for quantifying four typical oxidative stress biomarkers in human urine using solid-phase extraction (SPE) in conjunction with ultra performance liquid chromatography-tandem mass spectrometry (UPLC-MS/MS). The quantified biomarkers include L,L-dityrosine (diY), 8-hydroxy-2'-deoxyguanosine (8-OHdG), 8-hydroxyguanosine (8-OHG), and 4-hydroxynonenal mercapturic acid (HNEMA), which are markers of oxidative-stress-related damage in proteins, DNA, RNA, and lipids, respectively. To that end, we systematically optimized the MS parameters, SPE cartridge, and elution conditions of the method. Briefly, 0.2 mL of a urine sample was mixed with 0.8 mL of pure water, after which an internal-standard mixture was added. The four target analytes were enriched and purified using an HLB SPE cartridge. The diY and the other three compounds were eluted with 2% (volume fraction) methanol aqueous solution and methanol, respectively. The two groups of eluates containing different target analytes were separately injected onto an Acquity UPLC HSS T3 column (100 mm×2.1 mm, 1.8 μm) and gradient eluted using 0.05% (v/v) acetic acid aqueous solution and methanol. The target analytes were identified using both negative and positive electrospray ionization (ESI^-^ and ESI^+^) and multiple reaction monitoring (MRM) modes, and quantified using stable-isotope-labeled internal standards. The four typical oxidative-stress biomarkers exhibited good linearities within the mass concentration range of 0.01-100 μg/L, with correlation coefficients ≥0.9998, and limits of detection (LODs) and limits of quantification (LOQs) of 7-18 and 22-60 ng/L, respectively. The spiked recoveries of the target analytes at three levels (5, 10 and 50 μg/L) were 103.0%-105.6%(8-OHdG), 100.8%-104.2%(8-OHG), 97.2%-100.2%(diY) and 96.9%-106.0%(HNEMA), with intra-day precisions of between 1.6% and 5.2%. Moderate-to-strong matrix effects of between 42% and 137% were observed for each target analyte. The target compounds exhibited weak matrix effects of 99%-102% (8-OHdG), 97%-98% (8-OHG), 97%-106% (diY), and 94%-110% (HNEMA) after adjustment using the stable-isotope-labeled internal-standard method. The developed method was used to determine the abovementioned four typical oxidative stress biomarkers in 40 urine samples. All target compounds were detected in human urine at rates of 100%, with mass concentrations of 0.52-14.40 μg/L, 2.75-38.15 μg/L, 8.92-82.28 μg/L, and 1.74-575.29 μg/L recorded for 8-OHdG, 8-OHG, diY, and HNEMA, respectively, along with median values of 2.89, 12.36, 37.66, and 96.92 μg/L, respectively. The developed method is simple to operate, highly sensitive, and is very precise and accurate; consequently, it is suitable for determining the abovementioned four typical oxidative stress biomarkers in human urine.

氧化应激是指机体产生的活性氧与抗氧化防御系统失衡,从而引发一系列的氧化应激反应,最终导致细胞、蛋白质、DNA、RNA氧化应激损伤^[1⇓-3]^。氧化应激在人体生物监测领域发挥重要作用,通过分析尿液中氧化应激生物标志物的含量,能有效评估个体或群体对环境污染物暴露后的生理反应,对于早期发现污染物暴露的健康危害、评估污染物健康风险及影响具有重要意义。

常用于污染物暴露健康评估的氧化应激标志物有8-羟基脱氧鸟苷(8-OHdG)、8-羟基鸟苷(8-OHG)、羟基壬烯巯基尿酸(HNEMA)和双酪氨酸(diY)等,分别用于反映机体中DNA、RNA、脂质和蛋白质的氧化损伤^[4⇓⇓-7]^。目前,氧化应激生物标志物的检测方法主要包括酶联免疫吸附法(ELISA)^[8]^、高效液相色谱法(HPLC)^[9]^、气相色谱-质谱法(GC-MS)^[10]^和高效液相色谱-串联质谱法(HPLC-MS/MS)^[11]^。其中,ELISA只适用于某一类标志物的测定,且存在方法检出限较高和特异性弱等问题,进而影响测定结果的准确性^[8]^。GC-MS需要对目标分析物进行衍生化处理,存在操作繁琐且处理效率低等缺点^[10]^; HPLC存在DNA酶解不完全、干扰物质同时被洗出,导致测量值与真实值存在较大偏差等问题^[12]^。LC-MS虽然能够实现准确定量,但已报道的样品前处理方法多采用直接稀释法,如Hua等^[13]^用含0.1%乙酸的甲醇水溶液将尿液稀释3倍后进行测定,Yan等^[14]^用15 mmol/L乙酸铵将尿液稀释10倍后测定。上述前处理方法虽操作简单,但是不能有效去除尿液中的无机盐组分,不适合大批量的样品测定。此外,已有分析方法所涉及的氧化应激生物标志物类别较少^[15,16]^,缺乏同时检测可反映多系统氧化应激损伤标志物的分析方法,这也导致目前无法对污染物引发的机体氧化损伤进行全面评估。因此,亟需建立一种可同时检测DNA、RNA、蛋白质和脂质氧化应激生物标志物的方法,为环境污染物暴露所导致的氧化应激损伤提供更加充足的证据。

本研究基于超高效液相色谱-三重四极杆质谱法(UPLC-MS/MS)建立了人尿液中8-OHdG、8-OHG、HNEMA、diY 4种氧化应激生物标志物的分析方法。该法操作简便,灵敏度高,结果准确,精密度好,适于开展尿液中多种氧化应激生物标志物的分析测定。

## 1 实验部分

### 1.1 仪器、试剂与材料

I-Class Plus型超高效液相色谱-三重四极杆质谱仪(TQ-XS,美国Waters公司),Vortex-Genie2涡旋混匀器(美国Scientific Industries公司),AP-25D电子天平(美国Ohaus公司),24孔固相萃取装置(美国Supelco公司),Milli-IQ-7005纯水机(美国Milli-pore公司),MULTIVAP-48氮吹仪(美国Organomation公司)。

8 -OHdG(纯度>98%)购自美国Sigma-Aldrich公司;8-OHG(纯度>98%)购自美国Santa Cruz Biotechnology公司;diY(纯度>98%)购自美国Med Chem Express公司;HNEMA(纯度>98%)购自美国Good Laboratory Practice Bioscience公司;^13^C_12_-diY(纯度>98%)购自美国Cambridge Isotope Laboratories公司;^13^C-^15^N_2_-8-OHG(纯度>98%)购自广州佳途科技有限公司;^13^C-^15^N_2_-8-OHdG和D_3_-HNEMA购自加拿大Toronto Research Chemicals公司(纯度均>95%);甲醇、乙腈(MS级,德国Merck公司);乙酸(MS级,美国TIEDA公司); 实验用水由Milli-IQ-7005纯水机产生。Waters Oasis HLB固相萃取柱(3 mL, 60 mg)。

人群尿液样本采自北京某区,共40份,项目已获中国疾病预防控制中心环境与健康相关产品安全所伦理委员会批准(批准号:202105)。

### 1.2 实验条件

#### 1.2.1 色谱条件

色谱柱:Waters Acquity UPLC HSS T3色谱柱(100 mm×2.1 mm, 1.8 μm);流动相A: 0.05%(体积分数)乙酸水溶液;流动相B:甲醇;进样量:10 μL;柱温箱温度40 ℃;流速:0.25 mL/min;梯度洗脱:0~0.5 min, 99%A; 0.5~3.5 min, 99%A~35%A; 3.5~4.0 min, 35%A~0A; 4.0~8.5 min,0A; 8.5~8.6 min, 0A~99%A; 8.6~12.0 min,99%A。

#### 1.2.2 质谱条件

离子源:电喷雾电离(ESI)源;电离方式:8-OHdG、8-OHG和diY采用正离子模式(ESI^+^), HNEMA采用负离子模式(ESI^-^);扫描模式:多离子反应监测(MMR)模式;毛细管电压:2.46 kV;离子源温度:150 ℃;脱溶剂温度:500 ℃。4种目标物及其稳定同位素内标的详细质谱参数见表1。

**表1:** 4种氧化应激生物标准物及其稳定同位素内标的质谱参数

Compound	Retention time/min	Parent ion (*m/z*)	Daughter ions (*m/z*)	Cone voltages/V	Collision voltages/V
diY	2.94	361.2	254.1/315.1^*^	+25/+25	+22/+15
8-OHdG	3.48	284.1	140.0/168.0^*^	+18/+18	+30/+12
8-OHG	3.29	300.1	140.1/168.1^*^	+15/+15	+30/+15
HNEMA	5.45	318.4	162.0/171.1^*^/189.0	-18/-18/-18	-10/-20/-12
^13^C_12_-diY	2.94	373.3	266.2/327.3	+20/+20	+25/+15
^13^C-^15^N_2_-8-OHdG	3.48	287.1	171.1	+20	+15
^13^C-^15^N_2_-8-OHG	3.29	303.1	171.1	+20	+15
D_3_-HNEMA	5.42	321.1	165.1/189.1	-20/-20	-13/-13

diY: L,L-dityrosine; 8-OHdG: 8-hydroxy-2'-deoxyguanosine; 8-OHG: 8-hydroxyguanosine; HNEMA: 4-hydroxynonenal mercapturic acid. * Quantitative ion.

### 1.3 溶液配制

混合标准储备液(4 mg/L):准确称取4种氧化应激标志物标准品各1.000 mg至10 mL容量瓶中,甲醇定容后,将单标储备液放置。准确移取4种单标储备液1.0 mL至25 mL容量瓶中,甲醇定容,配制成质量浓度为4 mg/L的混合标准储备液。将混合标准储备液分装至2 mL安瓿瓶中,封口后于-80 ℃保存。

混合同位素内标储备液(4 mg/L):采用配制混合标准储备液相同的方法配制4种目标分析物的同位素内标储备液。使用时用水将混合同位素内标储备液稀释至1 mg/L。

系列标准溶液配制:用纯水将混合标准储备稀释为10、100、1000 μg/L的混合标准使用液,吸取适量混合标准使用液,配制成质量浓度为0.01、0.1、0.5、1、5、10、20、50、100 μg/L的系列标准溶液,内标添加量为10 μg/L。

### 1.4 样品前处理

样品准备:尿液样品由-80 ℃平衡至4 ℃,再平衡至室温。涡旋振荡尿液,取0.2 mL尿样于5 mL聚丙烯离心管中,依次加入0.8 mL水以及0.1 mL质量浓度为100 μg/L的混合同位素内标溶液(相当于10 ng内标),充分混匀后进行固相萃取。

固相萃取:使用3 mL甲醇和3 mL纯水活化和平衡Waters Oasis HLB固相萃取柱;将离心管中样品全部转移至固相萃取柱中,在不施加压力的情况下依靠自然重力过柱。样品过柱后,空气抽干2 min;用1 mL 2%(体积分数)甲醇水溶液进行洗脱,收集洗脱液,用于分析diY。空气抽干1 min,再用1.5 mL甲醇进行洗脱,氮吹至0.5 mL,加水0.5 mL,继续氮吹至0.5 mL,用初始流动相进行复溶至1 mL,用于分析8-OHdG、8-OHG和HNEMA。

## 2 结果与讨论

### 2.1 质谱参数优化

配制质量浓度为100 μg/L的目标分析物混合标准溶液,并对目标化合物及内标物质的质谱条件进行优化,包括母离子、子离子、锥孔电压和碰撞能量等参数。利用Intelli Start模式自动识别目标化合物的母离子和子离子。通过手动模式进行验证,以确定目标化合物的母离子和锥孔电压;最后对子离子和碰撞能量进行进一步的验证,确保子离子的信号强度最大化。具体参数见表1。

### 2.2 液相色谱条件优化

#### 2.2.1 色谱柱选择

比较了Acquity BEH C_18_ (100 mm×2.1 mm, 1.7 μm)、Acquity HILIC (100 mm×2.1 mm, 1.7 μm)、Acquity UPLC HSS T3 (100 mm×3.0 mm, 1.8 μm)和Acquity UPLC HSS T3 (100 mm×2.1 mm, 1.8 μm) 4种不同色谱柱对目标分析物的色谱分离效果。结果显示,当以甲醇-水作为流动相时,HILIC柱对4种目标分析物的保留及分离效果均较差,4种目标分析物的保留时间在1.0 min内。在实际测定时易受到基质干扰而影响定量结果。对比BEH C_18_和HSS T3色谱柱的分离效果,结果显示在相同流动相体系下,diY、8-OHdG和8-OHG经HSS T3柱分离后的质谱响应信号优于BEH C_18_柱。因此,实验选择HSS T3柱作为色谱柱。进一步比较两种不同规格的HSS T3柱对目标物的分离效果,发现当以100 mm×2.1 mm, 1.8 μm规格的T3色谱柱为分析柱时,HNEMA的色谱峰形更优,且其他3种目标分析物兼具更好的分离效果和更高的质谱响应信号。因此,在兼顾灵敏度和分离效果的前提下,实验选择以Acquity UPLC HSS T3柱(100 mm×2.1 mm, 1.8 μm)作为色谱柱进行目标分析物分离。

#### 2.2.2 流动相选择

确定色谱柱后,分别以甲醇-水和乙腈-水作为流动相,通过比较目标分析物的色谱峰形和质谱响应来评估两种流动相体系的洗脱效果,结果如图1所示。相比于甲醇,乙腈虽然具有更好的洗脱能力,但采用乙腈-水体系时4种氧化应激生物标志物的质谱响应信号明显低于甲醇-水体系。此外,考虑到甲醇成本更低,且毒性也更低。因此,实验最终选择甲醇-水流动相体系进行目标分析物梯度洗脱。

**图1:**
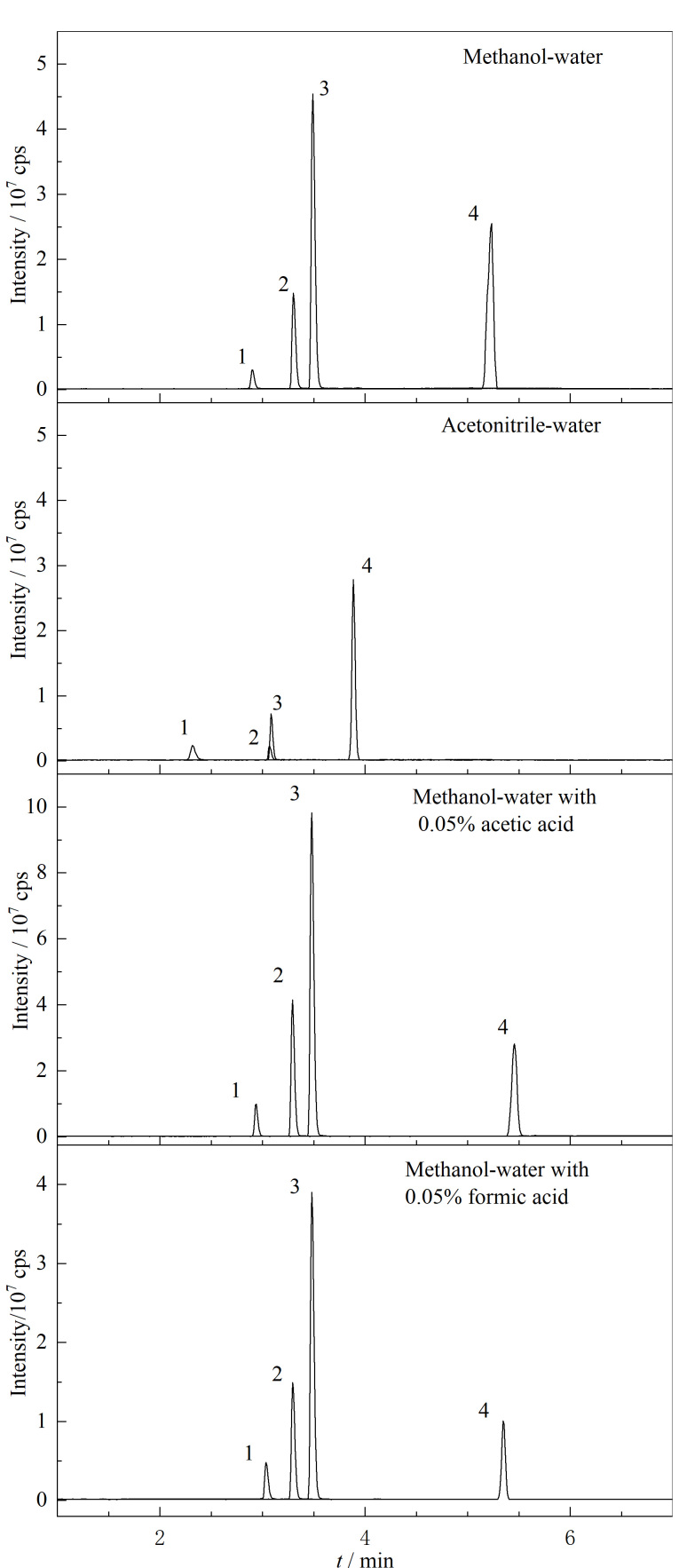
不同流动相条件下4种目标物(10 μg/L)的色谱图

向流动相中加入适量甲酸或乙酸,可以改善目标分析物在色谱柱上的分离效果,改善色谱峰峰形^[17]^。因此,分别向甲醇-水流动相体系中加入同体积的甲酸和乙酸,比较甲酸和乙酸对色谱分离的影响。当向流动相中添加0.05%(体积分数)乙酸后,diY、8-OHG和8-OHdG的质谱响应信号明显优于甲醇-纯水体系。其原因是乙酸的加入改变了目标分析物的离子化状态,使这些目标分析物更易电离。对比添加甲酸的色谱图,乙酸改善目标物在色谱柱上的分离效果明显优于甲酸,其峰形更加尖锐,质谱响应信号更好。进一步,为确定乙酸的最优添加比例,分别向流动相中加入0.05%和0.1%(体积分数)的乙酸,比较不同乙酸添加比例对色谱分离的影响。结果显示,当添加体积分数为0.1%乙酸时,HNEMA的质谱响应信号受到明显抑制。因此,实验最终选择0.05%(体积分数)乙酸水溶液为A相,甲醇为B相进行梯度洗脱。

### 2.3 前处理条件优化

#### 2.3.1 固相萃取柱选择

比较了Envi-carb (3 mL, 250 mg, Merck公司)、Oasis MAX(3 mL, 60 mg, Waters公司)和Oasis HLB 3种固相萃取柱对目标分析物的富集效果。分别用3 mL甲醇和3 mL纯水活化3种固相萃取柱,之后进行加标尿液的上样,并收集上样滤过液;分别使用3 mL甲醇淋洗Envi-carb和Oasis MAX柱, 3 mL纯水淋洗HLB柱,同时收集淋洗液;最后采用3 mL二氯甲烷、3 mL 2%(体积分数)甲酸甲醇和3 mL甲醇分别洗脱Envi-carb、Oasis MAX和Oasis HLB柱,同时收集洗脱液。通过对上样滤过液、淋洗液和洗脱液中的目标分析物进行分析,评估3种固相萃取柱的富集效果。结果显示,经MAX柱和HLB柱处理后的洗脱液中,均可检出8-OHdG、8-OHG和HNEMA的高强度质谱响应信号,这表明上述两种固相萃取柱对8-OHdG、8-OHG和HNEMA均有较好的富集效果。但上述两种固相萃取柱对diY的富集效果却不相同。经MAX柱处理后,上样滤过液中可见diY的高强度质谱响应信号,而淋洗液和洗脱液中未检出diY。这表明diY在上样过程中已随样品流失,无法在MAX柱上实现有效富集。当使用HLB柱处理样品时,diY在样品滤过液和淋洗液中都存在较高的质谱响应信号,而在洗脱液中无明显质谱响应信号,这说明diY可被HLB柱吸附,但吸附力较弱。而对于Envi-carb固相萃取柱而言,虽在其上样滤过液和淋洗液中均未检出目标分析物,但其对目标分析物的吸附能力太强,除二氯甲烷外,采用多种不同极性溶剂均无法实现目标分析物的有效洗脱。因此,综合考虑目标分析物在3种不同固相萃取柱上的吸附与洗脱,最终选择Oasis HLB柱进行固相萃取。

#### 2.3.2 上样条件优化

为增加diY在HLB柱上的富集效果,将6个不同来源的尿液分别用水稀释2倍、5倍和10倍,随后向稀释后的样品中加入等量diY内标,以内标的提取效率评估样品稀释对diY吸附的影响。结果显示,随着稀释倍数增加,diY内标的提取效率分别为35.3%、58.6%和59.5%。可见,对样品进行稀释可显著改善diY在HLB上的富集。其原因可能是尿液中可能含有与目标分析物产生竞争结合的基质干扰物,在尿液稀释后,其基质成分浓度降低,从而减少了对目标分析物的干扰,提高了diY的富集效果。

在获得稀释倍数后,继续使用不同体积分数(0.05%、0.1%、0.5%、1%、2%)的甲酸水溶液和乙酸水溶液对尿液进行稀释,考察不同酸性条件下diY在HLB柱上的富集情况。结果显示,采用甲酸水溶液稀释尿液能够增加diY在HLB柱上的富集;但同时HNEMA的加标回收率却随着甲酸体积分数增加而相应变低(如图2)。当甲酸体积分数为0.5%时,HNEMA的回收率仅为58%;而其余3种目标分析物的加标回收率为98%~103%。推测原因是甲酸能够促进D_3_-HNEMA分子中氘原子与质子发生交换反应。随着时间的推移,氘的交换过程逐渐进行,导致D_3_-HNEMA分子丢氘,形成更易于离子化的形式,从而使其质谱信号不断增加。而用乙酸水溶液进行样品稀释后,既不能增加diY在HLB柱上的保留,也对4种目标分析物的加标回收率没有造成影响(4种目标分析物的加标回收率为93%~105%)。因此,实验最终选择用水将样品稀释5倍后进行固相萃取。

**图2:**
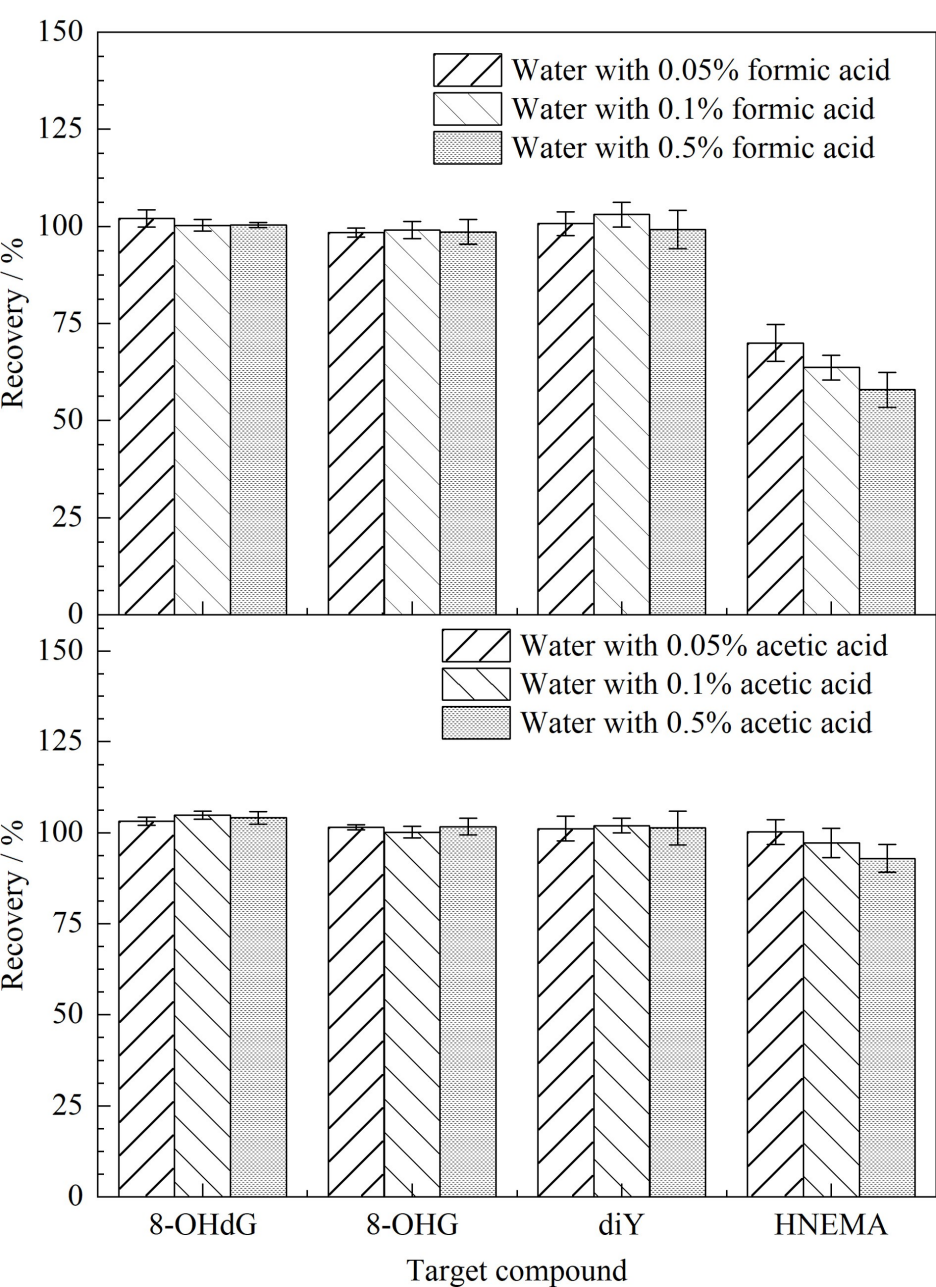
采用不同稀释溶剂时4种目标物的加标回收率(*n*=3)

为进一步提高diY在HLB柱上的保留,分别用水和甲醇作为基质配制内标使用液,并在两种配制方法下向尿液中加入等体积使用液,比较diY在两种加标方式下的富集效果。结果显示,与甲醇相比,用水配制内标使用液并添加内标后,diY的提取效率从59%提高至70%。其原因是使用甲醇配制的内标使用液在加标过程中引入了更多的甲醇基质,导致上样时样品极性变弱,不利于diY在HLB柱上保存。因此,实验最终选择以水为基质配制内标使用液并进行内标添加。

#### 2.3.3 洗脱溶剂选择

考察了不同体积分数的甲醇水溶液对diY的洗脱效果。向尿液中加入10 ng混合内标,样品过HLB柱后,用1 mL含1%、2%、5%、10%甲醇的水溶液进行第一步洗脱。结果显示,diY内标在洗脱液中的色谱峰面积随甲醇比例的提高而增加。但当甲醇体积分数为5%时,洗脱液中8-OHG内标的色谱峰峰面积显著增加。原因是随着甲醇体积分数增加,洗脱液的极性逐渐变弱。弱极性试剂在反相吸附柱上的洗脱强度更大,进而使样品中的8-OHG被洗出而出现样品损失,进一步影响8-OHG的提取效率。因此,实验最终选择2%体积分数的甲醇水溶液进行第一步洗脱。

完成diY洗脱优化后,继续比较了甲醇、乙腈、乙酸乙酯、二氯甲烷4种洗脱溶剂对8-OHdG、8-OHG和HNEMA的洗脱效果。3种物质的加标回收率如图3a所示,以二氯甲烷为洗脱溶剂时,3种目标分析物的加标回收率结果明显低于其他3类洗脱溶剂;而经甲醇、乙腈、乙酸乙酯处理后3种目标分析物的加标回收率差异不明显。为获得较优的洗脱条件,向处理前的实际尿液中加入3种目标分析物标准品,采用本方法进行前处理后进样,得到3种目标分析物定量离子峰面积;同时,对相同的实际尿液进行前处理,并向处理后的基质中加入等量标准品,再次进样后得到3种目标分析物的定量离子峰面积。通过两次峰面积的比值,获得了3种不同洗脱溶剂条件下目标分析物的提取效率。结果如图3b,以甲醇为洗脱溶液时各目标分析物的提取效率为102.9%、100.2%、97.1%,高于乙腈和乙酸乙酯。因此,最终实验选择以甲醇作为第二步洗脱溶剂。

**图3:**
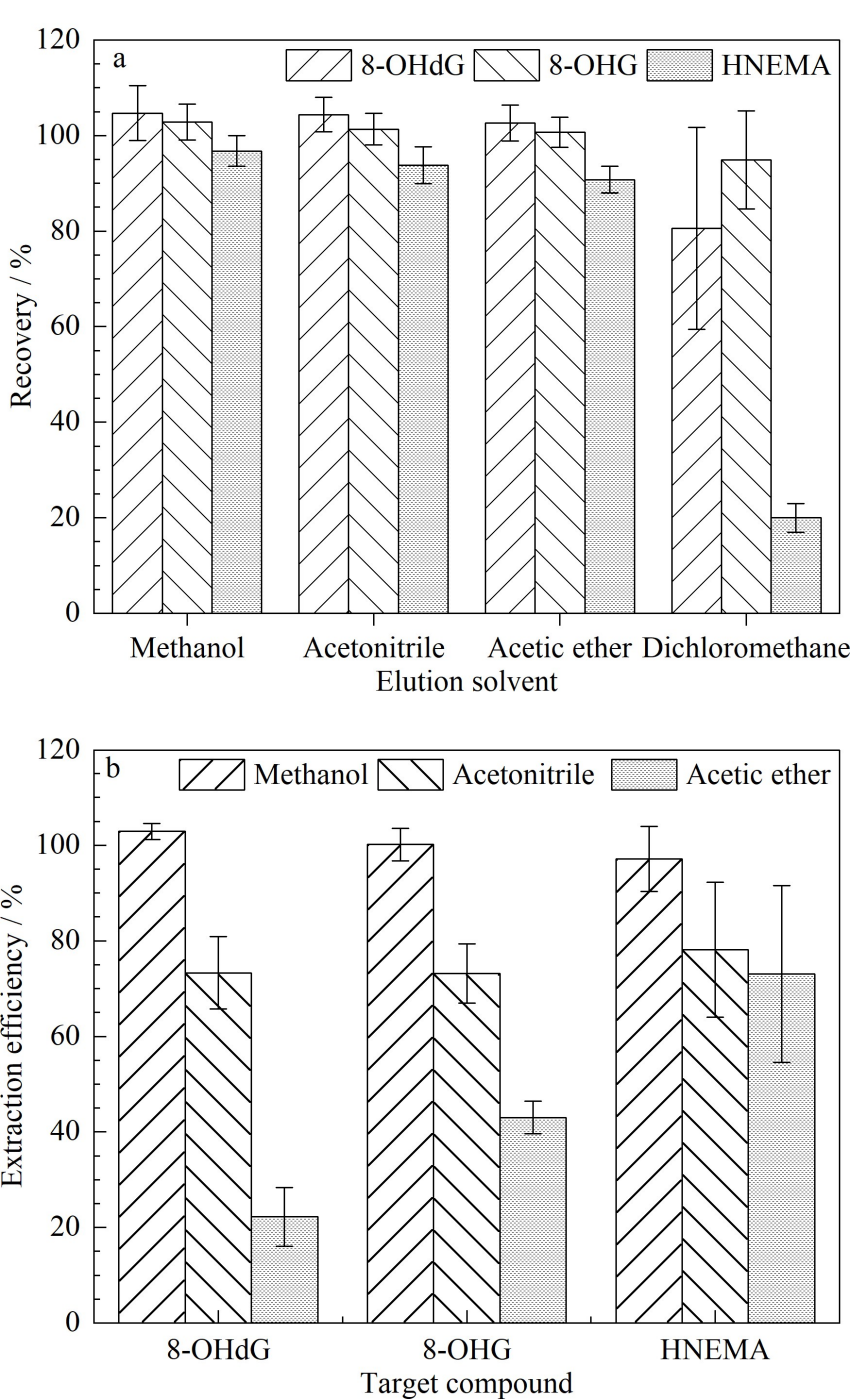
目标物在不同洗脱溶剂条件下的(a)回收率和(b)提取效率(*n*=6)

### 2.4 基质效应

分析方法的精密度和准确性可能受到样本中复杂基质的影响,因此应该对方法的基质效应进行评估,进一步判断整个处理过程是否需要用内标进行校正。按照文献[18,19]的方法,选用6个不同来源的尿液,按照本研究的前处理方法进行处理,以6个尿液样品检测前的溶液为基质配制尿液基质标准溶液,同时以水为基质配制相同浓度的标准溶液。以两条标准曲线斜率的比值来评估基质效应。ME为80%~120%时为弱基质效应,ME为50%~80%或120~150%时表示中基质效应,ME为<50%或>150%时表示强基质效应。结果如图4a所示,在6个不同尿液中,8-OHdG和diY的ME分别为58%~86%和109%~137%,表现为从弱基质到中基质效应;而8-OHG和HNEMA的ME分别为48%~63%和42%~60%,表现为从中等基质效应到强基质效应。上述结果表明在定量过程中,需要使用同位素内标进行校正,以降低基质效应对定量结果的影响。图4b显示,经同位素内标校正后,8-OHdG、8-OHG、diY和HNEMA的基质效应分别为99%~102%、97%~98%、97%~106%和94%~110%,均表现为弱基质效应。

**图4:**
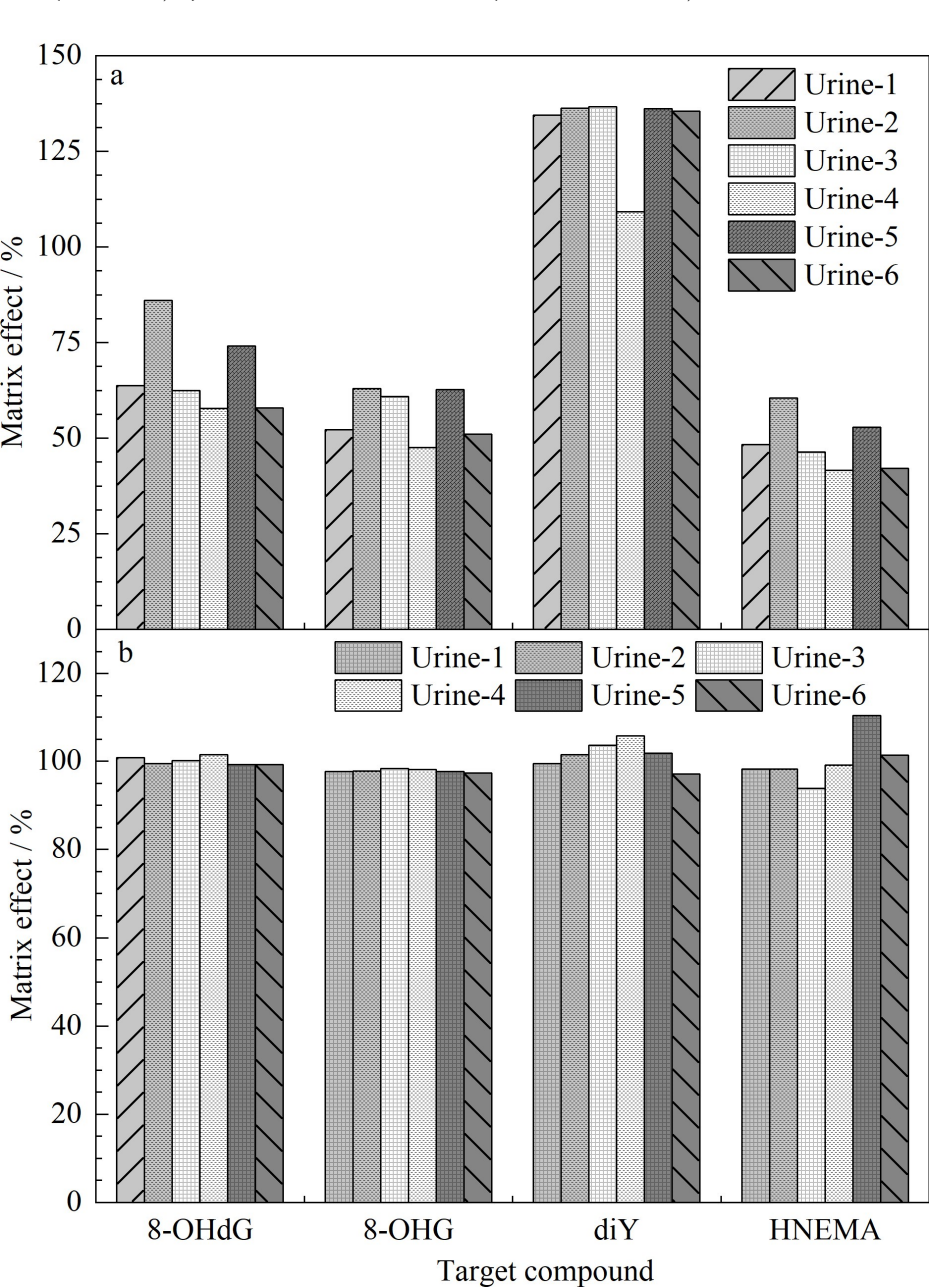
不同尿液中4种目标分析物的基质效应

### 2.5 方法学评价

#### 2.5.1 线性范围、检出限与定量限

配制系列标准溶液,在本研究所述色谱条件和质谱条件下进行测定。以目标分析物及其相应同位素内标的定量离子的峰面积之比为纵坐标,以目标分析物的质量浓度为横坐标绘制标准曲线,标准曲线拟合时采用1/*x*进行曲线加权,4种目标分析物在标准曲线线性范围内线性关系良好,相关系数(*r*)均≥0.9998;以3倍信噪比(*S/N*=3)计算检出限(LOD), 10倍信噪比(*S/N*=10)计算定量限(LOQ)。4种目标分析物的线性范围、相关系数、LOD和LOQ见表2。

**表2:** 4种目标分析物的线性范围、相关系数、检出限和定量限

Compound	Linear range/ (μg/L)	*r*	LOD/ (μg/L)	LOQ/ (μg/L)
diY	0.01-100	0.9999	0.018	0.060
8-OHDG	0.01-100	0.9999	0.011	0.037
8-OHG	0.01-100	0.9998	0.010	0.034
HNEMA	0.01-100	0.9999	0.007	0.022

#### 2.5.2 回收率与精密度

选择6个实际尿液样品进行低(5 ng/mL)、中(10 ng/mL)、高(50 ng/mL)3个水平的加标回收试验。在同一自然日中,对同一实际尿样进行6次平行测定,评估方法日内精密度。详细结果如表3所示,8-OHdG、8-OHG、diY和HNEMA的加标回收率分别为103.0~105.6%、100.8%~104.2%、97.2%~100.2%、96.9%~106.0%,日内精密度为1.6%~5.2%。

**表3:** 4种目标分析物在低、中、高3个水平下的加标回收率和日内精密度(*n*=6)

Compound	Low			Medium			High		Intra-day RSD/%
	Recovery/%	RSD/%		Recovery/%	RSD/%		Recovery/%	RSD/%	
diY	100.2	1.4		97.8	0.8		97.2	0.9	1.7
8-OHdG	105.6	1.4		104.9	1.4		103.0	0.8	1.6
8-OHG	104.2	0.7		100.9	0.9		100.8	1.1	1.8
HNEMA	106.0	4.1		102.0	4.5		96.9	2.2	5.2

### 2.6 方法比较

如表4所示,目前已有的氧化应激标志物分析方法主要涉及2~3类氧化应激标志物的检测,缺少可以同时测定DNA、RNA、脂质和蛋白质4类氧化应激标志物的分析方法;其次,本方法的LOD明显低于其他方法;最后,本方法的样本用量也处于较低水平,虽然文献[13]和文献[21]样本用量更少,但上述方法中目标分析物的加标回收率分别为77%~101%和88.7%~95.4%,低于本研究所获得的加标回收率。因此,与其他研究相比,本方法在增加检测类别的同时也保证了方法的灵敏度和准确度,可为人群尿液样本中不同类型氧化应激标志物的同时检测提供可靠的技术支持。

**表4:** 本方法与文献报道的人尿液中氧化应激标志物检测方法的比较

No.	Oxidative stress biomarker				*n*	Extraction method	Instrumental analysis method	LOD/ (μg/L)	Sample volume/μL	Ref.
	DNA	RNA	Lipid	Protein						
1	√	√	/	√	3	/	HPLC-MS/MS	0.058-0.093	/	[11]
2	√	/	√	√	7	derivatization-SPE	HPLC-MS/MS	0.01-0.03	500	[20]
3	√	/	/	√	4	dilution	UPLC-MS/MS	0.007-0.083	50	[13]
4	√	/	√	/	4	SPE	HPLC-MS/MS	0.008-0.03	100	[21]
5	√	√	/	/	2	SPE	UPLC-MS/MS	/	200	[22]
6	√	/	√	/	2	SPE	UPLC-MS/MS	0.01-0.02	500	[23]
7	√	√	√	√	4	SPE	UPLC-MS/MS	0.007-0.018	200	this study

*n*: the total number of the target compounds in the method; √: mentioned; /: not mentioned.

### 2.7 实际样品测定

采用本方法测定了40份尿液样本中4种氧化应激生物标志物。结果显示,8-OHdG、8-OHG、diY和HNEMA均可检出,检出率为100%;检出质量浓度范围分别为0.52~14.40 μg/L、2.75~38.15 μg/L、8.92~82.28 μg/L和1.74~575.29 μg/L,中位值分别为2.89、12.36、37.66和96.92 μg/L。Yan等^[14]^测定了广东省清远地区垃圾拆解地附近居民尿液中8-OHdG、8-OHG、diY的含量水平,发现3种氧化应激标志物的检出率分别为91.3%、99.0%、100%,检出质量浓度的中位值分别为8-OHdG 2.35 μg/L、8-OHG 14.50 μg/L和diY 55.30 μg/L。与本研究相比,其较低的检出率可能是由于该研究采用直接稀释法处理尿液,导致方法检出限高于本研究所致;而较高的检出浓度可能是由于电子拆解地居民受到了多溴联苯醚、短链氯化石蜡等污染物暴露,进而导致氧化应激水平升高。Wu等^[21]^测定得到的口腔黏膜炎病例队列人群尿液中8-OHdG(22.08 μg/L)和HNEMA(100.15 μg/L)的中位浓度也高于本研究,其原因可能是本研究所检测人群为一般人群,其氧化应激水平一般低于病例人群。Martinez等^[20]^测定了美国纽约人群尿液样本中的diY,其中位质量浓度(0.99 μg/L)低于本研究,而8-OHdG中位质量浓度(9.18 μg/L)高于本研究,这可能是因为不同的环境因素、地理与人种因素引起的差异。

## 3 结论

本研究采用超高效液相色谱-串联质谱法建立了人尿液中4种氧化应激生物标志物的测定方法。对仪器和前处理条件进行了优化,从方法学指标和基质效应等方面对方法进行了评估,并进行了实际样品的测定。本方法具有操作简单、检测结果准确、检出限低和重复性好的优点,适用于人尿液中4种氧化应激标志物的分析测定。
